# Primary aortic coarctation repair in adolescents and adults

**DOI:** 10.1016/j.xjse.2024.100034

**Published:** 2024-11-05

**Authors:** Matthew A. Thompson, William C. Frankel, Jonathan Putnam, Holliann Willekes, Benjamin Kramer, Ashley M. Lowry, Tara Karamlou, Patcharapong Suntharos, Joanna Ghobrial, Lars G. Svensson, Eugene H. Blackstone, Eric E. Roselli

**Affiliations:** a Department of Thoracic and Cardiovascular Surgery, Heart, Vascular, and Thoracic Institute, Cleveland Clinic, Cleveland, Ohio; b Department of Quantitative Health Sciences, Lerner Research Institute, Cleveland Clinic, Cleveland, Ohio; c Department of Pediatric Cardiology, Pediatrics Institute, Cleveland Clinic, Cleveland, Ohio; d Department of Cardiovascular Medicine, Heart, Vascular, and Thoracic Institute, Cleveland Clinic, Cleveland, Ohio

**Keywords:** endovascular, extra-anatomic bypass, frozen elephant trunk, hypertension, stent graft

## Abstract

**Objective:**

Optimal management of primary aortic coarctation in adolescents and adults remains controversial. We assess early and late mortality of repair, longitudinal antihypertensive regimens, and freedom from reintervention for repair-related complications.

**Methods:**

From January 1, 1999, to July 1, 2023, 110 adolescents or adults, mean age 39 ± 16 years, underwent primary aortic coarctation repair at Cleveland Clinic. Patients were grouped by repair strategy: stented (60, 55%), grafted (interposition graft or extra-anatomic bypass; 36, 33%), or other (14, 13%). Longitudinal antihypertensive regimens were assessed parametrically. Freedom from reintervention and survival were assessed by the Kaplan–Meier method. Median follow-up for survival was 9.2 years.

**Results:**

One patient (0.91%) died in-hospital. Major morbidity included stroke (2/107, 1.9%), tracheostomy (2/107, 1.9%), acute renal failure requiring dialysis (1/108, 0.93%), iatrogenic aortic dissection (1/107, 0.93%), and vascular access complication (1/56, 1.8%). Prevalence of patients requiring zero antihypertensive medications postoperatively peaked within 2 years. Twelve-year freedom from reintervention was 69% in the stented group and 97% in the grafted group (*P* log-rank = .003). Most reinterventions were for in-stent restenosis of bare metal stents. Overall freedom from reintervention at 1, 5, and 12 years was 91%, 80%, and 76%, respectively. Overall survival was 98%, 95%, and 80% at 5, 10, and 14 years, respectively.

**Conclusions:**

A tailored approach to coarctation repair in adolescents and adults using stented repair when anatomically feasible yields excellent short-term outcomes, but patients require persistent hypertension monitoring and have lower than expected long-term survival. Covered stents or stent grafts are preferred to bare metal stents, which are subject to in-stent restenosis.

## Graphical Abstract

**Figure:**
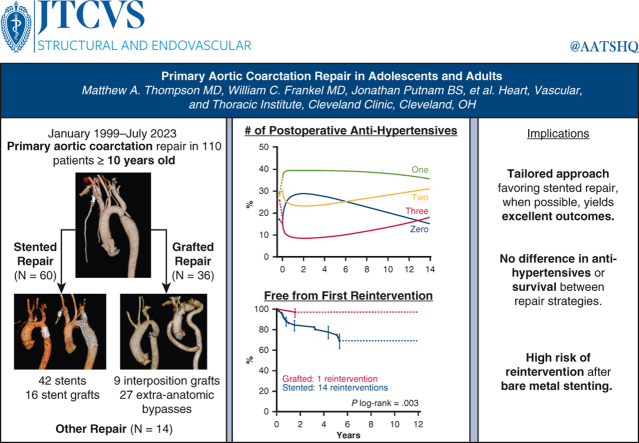

**Figure:**
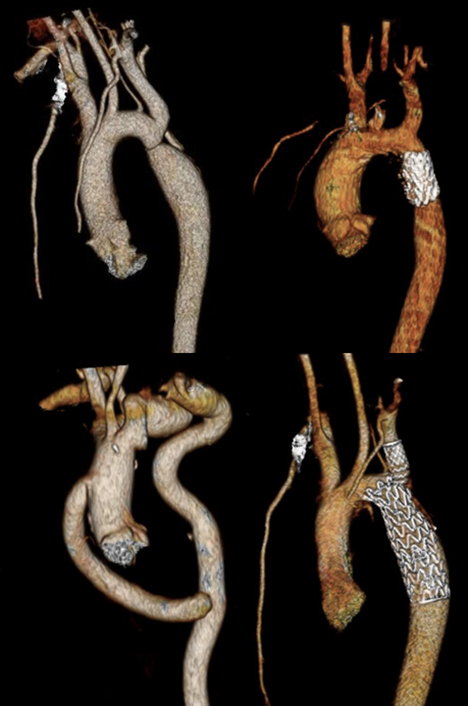
Coarctation (top left) repaired by stent, stent graft, and extra-anatomic bypass (clockwise).

**Central Message**

Stented and grafted repair of aortic coarctation in adolescents and adults have excellent short-term outcomes but high risk of reintervention after bare metal stenting and compromised late survival.

**Perspective**

In adolescents and adults with primary aortic coarctation, we increasingly use stented repair, preferably with a covered stent or stent graft, in patients with amenable anatomy without concomitant cardiovascular lesions. Otherwise, we perform grafted repair, usually extra-anatomic bypass. Hypertension screening is recommended long term, even in patients who are initially normotensive after repair.

Although coarctation of the aorta is often diagnosed before birth or early in life, with many patients undergoing surgical repair in childhood, a subset of patients present in adolescence or adulthood.^1^ In these patients, choosing the repair strategy is a complex decision tailored to their comorbidities, aortic arch and coarctation anatomy, and presence of concomitant cardiovascular lesions. We previously reported outcomes of coarctation repair in older patients at our center from 1999 to 2011 in a heterogeneous cohort that included primary coarctation, recurrent coarctation, and postcoarctation complications as indications for repair.^1^ Over the last decade, there has been robust evolution of endovascular techniques for treatment of the thoracic aorta, and we have gained greater experience treating primary coarctation at our center. We sought to provide an updated analysis focused on primary coarctation repair techniques, outcomes with longer follow-up, and insight into our clinical decision-making process.

## Material and Methods

### Patients

From January 1, 1999, to July 1, 2023, 110 adolescents and adults (mean age 39 ± 16 years, 62% male) underwent primary aortic coarctation repair at Cleveland Clinic (Table 1). Eighteen patients with a history of cardiac surgery were included because they had not undergone previous coarctation repair (Table E1). Exclusion criteria were age less than 10 years, pseudocoarctation, and coarctation remote from the aortic isthmus. Median peak transcoarctation gradient was 36 mm Hg. All data were approved for use in research by the Cleveland Clinic Institutional Review Board on February 8, 2023, with patient consent waived (IRB #23-168).

**Table 1:** Patient characteristics

Characteristic	n∗	Summary (N = 110)
Comorbidities		
Age (y)	110	39 ± 16
Male	110	68 (62)
Hypertension	110	97 (88)
No. of antihypertensives	107	
0		22 (21)
1		33 (31)
2		27 (25)
≥3		25 (23)
Smoking	110	36 (33)
Dyslipidemia	110	25 (23)
Atrial fibrillation	110	10 (9.1)
Congestive heart failure	110	10 (9.1)
Coronary artery disease	110	9 (8.2)
Myocardial infarction	110	8 (7.3)
Stroke	110	7 (6.4)
Diabetes	110	5 (4.5)
Cardiovascular morphology		
Concomitant congenital cardiac anomalies	110	77 (70)
Bicuspid aortic valve		55 (50)
Hypoplastic aortic arch		14 (13)
Patent ductus arteriosus		7 (6.4)
Ventricular septal defect		5 (4.5)
Interrupted aortic arch		4 (3.6)
Hypoplastic abdominal aorta		4 (3.6)
Pan-hypoplastic aorta		4 (3.6)
Other		17 (15)
Zone 1 diameter (mm)	68	25 ± 5.2
Zone 2 diameter (mm)	74	22 ± 4.6
Left subclavian artery diameter (mm)	79	15 ± 4.3
Peak transcoarctation gradient (mm Hg)	87	36 (20-57)
Concomitant aneurysm	108	23 (21)
Root		14 (13)
Ascending		16 (15)
Arch		1 (0.93)
Descending		5 (4.6)

∗ Number with data available.

### Coarctation Repair Techniques

**Figure 1:**
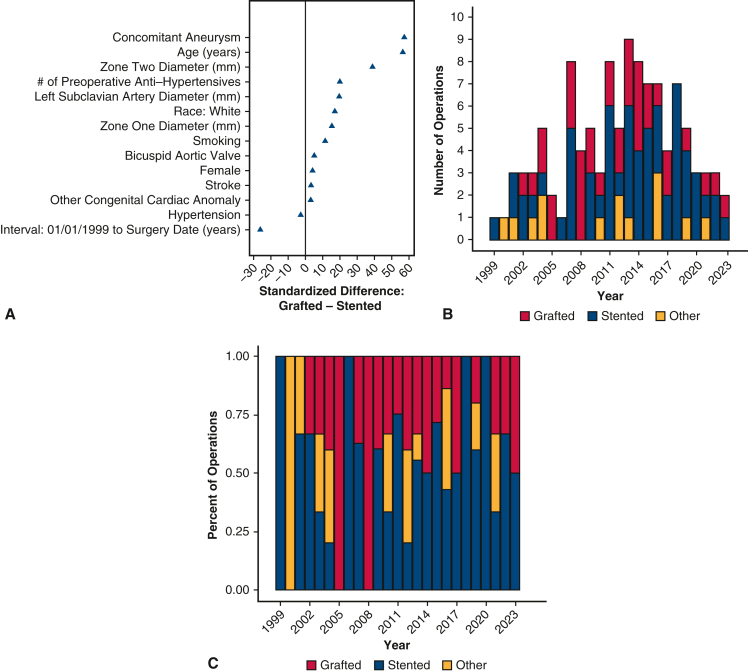
Standardized differences of selected variables between stented and grafted groups (A). Annualized number of primary aortic coarctation repairs stratified by repair strategy (B). Percentage of primary aortic coarctation repairs stratified by repair strategy (C).

Patients underwent 1 of 3 general repair strategies: stented (60, 55%), grafted (36, 32%), or other (14, 13%) (Table 2). Compared with those in the grafted group, patients in the stented group were younger and less likely to have a concomitant thoracic aortic aneurysm. The number of preoperative antihypertensives was not significantly different between the stented and grafted groups (Figure 1, *A*, and Table E2). There was a trend toward stented repair later in the study period (Figure 1, *B* and *C*).

**Table 2:** Operative details

Repair strategy	n∗	Summary (N = 110)
Stented	110	60 (55)
Device type	58	
Balloon-expandable stent		42 (72)
Bare metal		31 (53)
Covered		6 (10)
Bare metal + covered		5 (8.6)
Self-expanding stent graft		15 (26)
Diameter (mm)	15	28 (26-34)
Length (mm)	15	100 (100-140)
Bare metal + stent graft		1 (1.7)
Left subclavian artery management	58	
Native flow		44 (76)
Left common carotid–subclavian bypass		11 (19)
Revascularization with stent		3 (5.2)
Grafted	110	36 (33)
Extra-anatomic bypass	36	27 (75)
Sternotomy		22 (61)
Thoracotomy		5 (14)
Graft diameter (mm)	26	20 (18-22)
Interposition graft	36	9 (25)
Sternotomy		1 (2.8)
Thoracotomy		8 (22)
Graft diameter (mm)	7	20 (18-22)
Other	110	14 (13)
Frozen elephant trunk		6 (5.5)
End-to-end		5 (4.5)
Patch angioplasty		2 (1.8)
Conventional elephant trunk		1 (0.9)

∗ Number with data available.

#### Stented repairs

**Figure 2:**
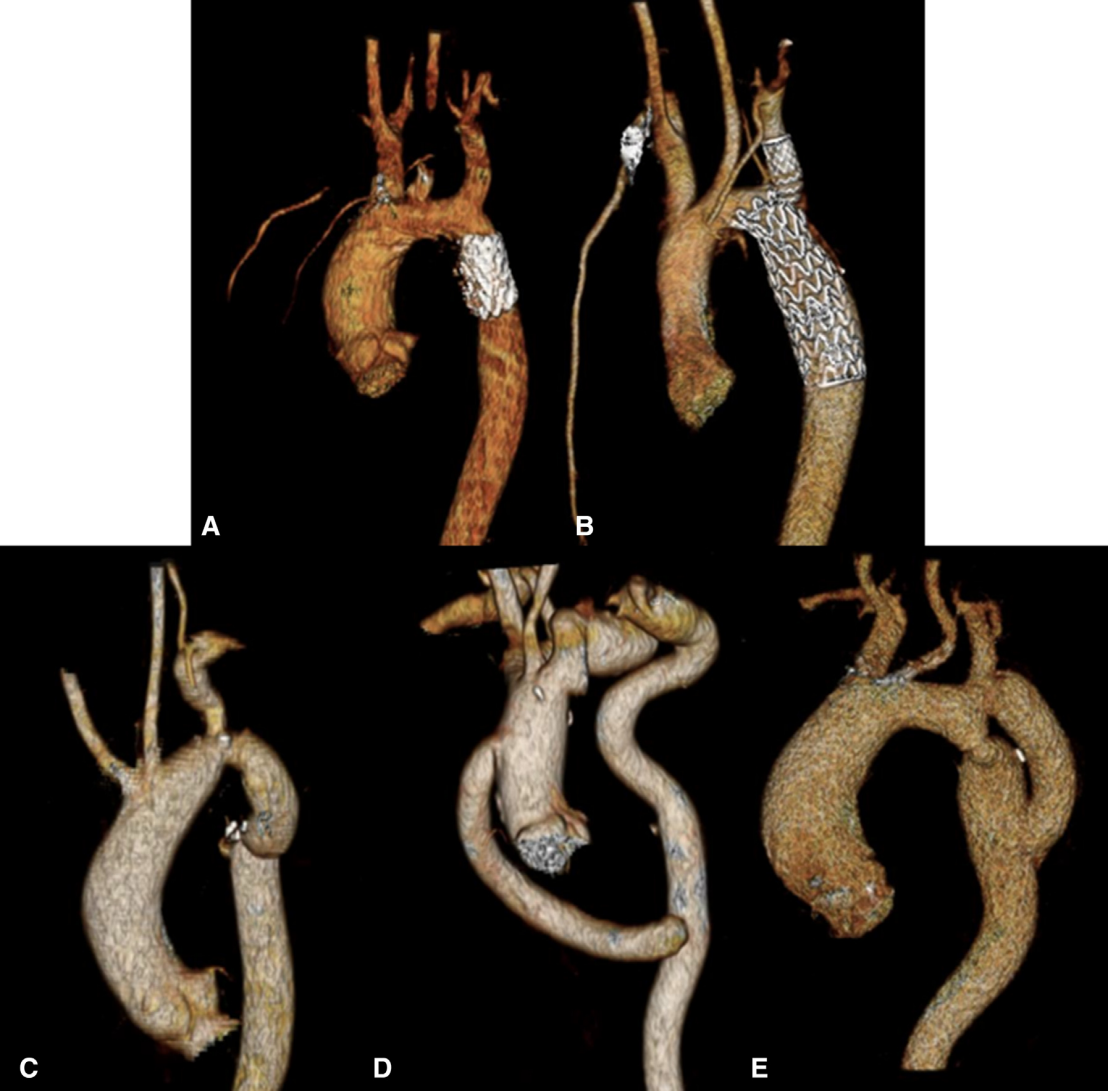
Postoperative computed tomography of patients with primary coarctation repaired via balloon-expandable stent (A), thoracic branched endoprosthesis (B), interposition graft (C), ascending–descending extra-anatomic bypass (D), and arch–descending extra-anatomic bypass (E).

Sixty patients underwent stented repair with balloon-expandable stents (42, 70%), stent grafts (15, 25%), or both (1, 1.7%) (Figure 2, *A* and *B*). Device type was unknown or a device not used in 1 patient each. A single device was used in 51 patients, 2 devices were used in 12 patients, and 4 devices were used in 1 patient. Technical success was defined as a transcoarctation gradient less than 15 mm Hg by direct measurement after device deployment. One patient who underwent an endovascular procedure had a concomitant patent ductus arteriosus ligation.

#### Grafted repairs

Thirty-six patients underwent grafted repair, including 9 interposition grafts and 27 extra-anatomic bypasses (Figure 2, *C-E*). Interposition grafting was performed via left thoracotomy in 8 patients or median sternotomy in 1 patient who underwent concomitant ascending aortic and arch aneurysm replacement. Extra-anatomic bypass was performed via left thoracotomy in 5 patients and median sternotomy in 22 patients. Nineteen patients who underwent extra-anatomic bypass via median sternotomy had concomitant cardiovascular procedures, including 14 aortic valve procedures, 11 ascending aortic repairs, 4 aortic root repairs, 3 coronary artery bypasses, 2 mitral valve procedures, 1 aortic arch replacement, and 1 tricuspid valve repair. In most cases, we performed ascending–descending bypass by tunneling an 18- to 22-mm graft retrocavally through the oblique sinus. In 3 patients, we performed arch–descending bypass via thoracotomy. All grafted repairs used partial left atrial–femoral bypass or full cardiopulmonary bypass and were cooled to 30°C to 32°C. Median cardiopulmonary bypass times were 69 minutes for interposition grafting and 133 minutes for extra-anatomic bypass.

#### Other repairs

Fourteen other repairs included 6 frozen elephant trunks, 5 coarctation excisions with direct end-to-end anastomosis, 2 patch angioplasties, and 1 conventional elephant trunk. Apart from frozen elephant trunk, these repairs were exclusively performed before 2013. Five patients who underwent frozen elephant trunk had concomitant procedures.

### End Points

**Figure E1:**
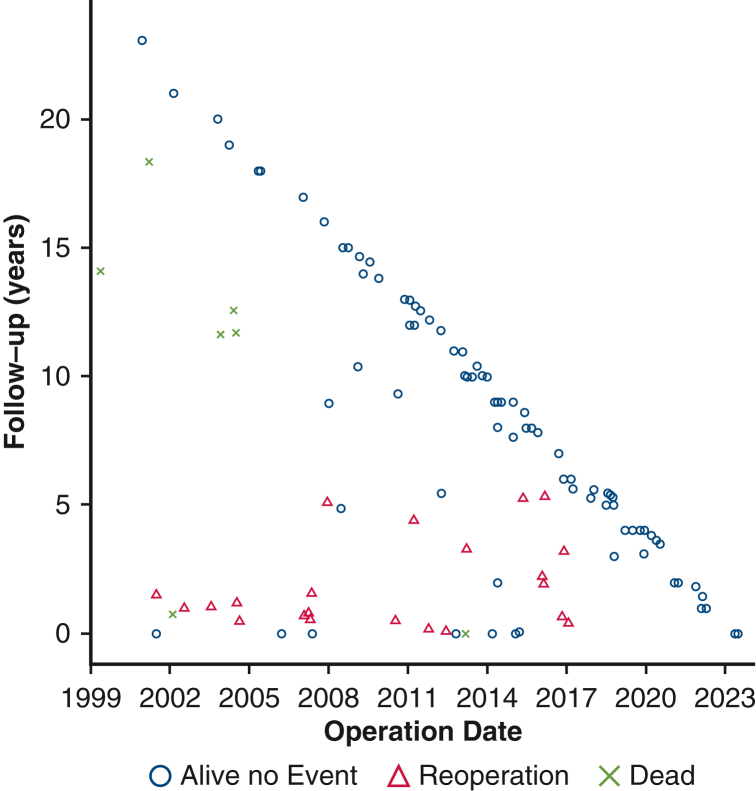
Goodness of follow-up for survival.

Operative mortality and major morbidity were defined using the Society of Thoracic Surgeons Adult Cardiac Surgery Database definitions. Time-related end points were (1) number of antihypertensives; (2) reintervention for in-stent restenosis, recoarctation, or another repair-associated complication; and (3) death. Secondary end points were postoperative in-hospital morbidity and mortality. The number of antihypertensives was manually extracted from the electronic medical record using postoperative primary care and cardiology visits. We conducted cross-sectional follow-up by telephone beginning in November 2023 to query patients on their need for cardiovascular reinterventions and to confirm vital status. Median follow-up was 6.4 years for antihypertensives, with 638 records available for 93 patients, and 9.2 years for survival (Figure E1).

### Data Analysis

Statistical analyses were performed with SAS version 9.4 (SAS Institute, Inc) and R version 4.3.1 (R Foundation for Statistical Computing). Continuous variables are summarized as mean ± SD or 15th, 50th, and 85th percentiles when data were skewed and categorical variables as frequency and percent. Comparisons were made using the Wilcoxon rank-sum test for continuous variables and the chi-square test, and the Fisher exact test was used when the frequency is less than 5 for categorical variables. Preoperative and postoperative transcoarctation gradients were not compared between stented and grafted groups due to heterogeneity in method of measurement and patient activity state.

A nonlinear longitudinal decomposition mixed-effects model was used to characterize temporal patterns in the number of postoperative antihypertensives with patients were entered as random effects (SAS PROC NLMIXED).^2^^,^^3^ The proportion of patients taking zero, 1, 2, or 3 or more antihypertensives was estimated by averaging patient-specific profiles. Freedom from reintervention and survival were assessed nonparametrically by the Kaplan–Meier method. Comparisons were made using the log-rank test.

## Results

### Technical Success

Median peak transcoarctation gradient was zero mm Hg postoperatively. Among patients who underwent stented repair, postoperative transcoarctation gradient was known for 51 (85%). Technical success was achieved in 49 patients (96%). Failures occurred in 1 patient who had a persistent peak gradient of 20 mm Hg after bare metal stenting and 1 patient whose endovascular procedure was aborted because of the inability to traverse the coarctation with a guidewire. The latter patient underwent subsequent repair at an outside hospital. Postoperative transcoarctation gradient was rarely reported for patients who underwent grafted repair.

### Hospital Outcomes

There was 1 operative death (0.9%) in a 49-year-old man who underwent frozen elephant trunk repair with concomitant valve-sparing root replacement. He developed postcardiotomy shock, left the operating room on venoarterial extracorporeal membrane oxygenation, and died of multiorgan system failure on postoperative day 7.

There were 2 instances of stroke (1.9%): 1 in a patient admitted with biventricular heart failure who underwent stented repair and developed a left middle cerebral artery stroke postprocedure, and 1 in a patient who underwent frozen elephant trunk repair. Two patients (1.2%) required tracheostomy. There was 1 (0.9%) iatrogenic aortic dissection and vascular access complication in the stented group and 1 instance of new dialysis after frozen elephant trunk repair. No patient required a pacemaker or experienced lower-extremity paralysis due to spinal cord ischemia. Median postoperative length of stay was 1 day for the stented group and 6 days for the grafted group (*P* < .0001) (Table 3).

**Table 3:** Hospital outcomes

Outcome	n∗	Frequency (N = 110)
Peak transcoarctation gradient (mm Hg)	67	0 (0-7)
Operative length of stay (d)	107	5 (1-8)
Hospital death	110	1 (0.91)
Stroke	107	2 (1.9)
Tracheostomy	108	2 (1.9)
Iatrogenic aortic dissection	107	1 (0.93)
New dialysis	108	1 (0.93)
Vascular access complication	56†	1 (1.8)

∗ Number with data available.

† N = 60 patients who underwent stented repair.

### Antihypertensive Medications

**Figure 3:**
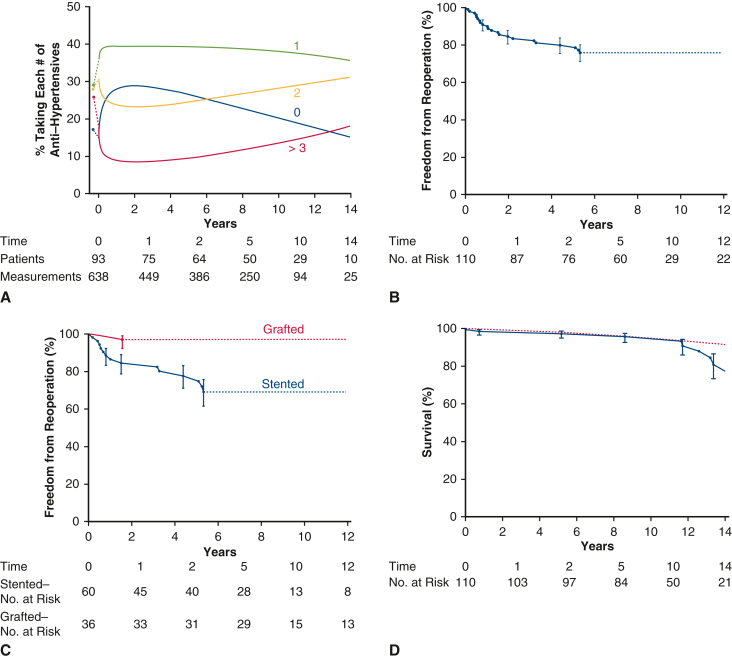
Number of antihypertensives after a stented or grafted primary coarctation repair (A). Freedom from first reintervention for coarctation-associated complications overall (B) and stratified by stented (*blue*) or grafted (*red*) repair (*P* log-rank = .003, C). Overall survival. For reference, *dotted line* represents survival of an age, sex, and race-matched US population (D).

The prevalence of patients taking zero or 1 antihypertensive peaked within 2 years and then declined throughout the remainder of follow-up (Figure 3, *A*). Conversely, prevalence of patients taking 2 and 3 antihypertensives increased from 2 to 14 years postoperatively. Despite an initial decrease in antihypertensives after repair, by 14 years, patients’ antihypertensive needs were similar to their preoperative regimen.

### Reinterventions

Twenty-two patients (14 stented, 1 grafted, 7 other) underwent reintervention. Reinterventions were for in-stent restenosis (13, 73%), repair-associated aneurysm (5, 23%), recoarctation (3, 14%), type B dissection (2, 9%), endoleak (1, 4.5%), and graft infection (1, 4.5%). In the stented group, all but 1 reintervention was for in-stent restenosis in patients who received a bare metal stent. In the grafted group, 1 patient underwent reintervention for an infected extra-anatomic bypass graft. Nineteen reinterventions were performed endovascularly, 2 open and 1 hybrid, with no operative deaths. Freedom from reintervention for the overall cohort at 1, 5, and 12 years was 91%, 80%, and 76%, respectively (Figure 3, *B*). Twelve-year freedom from reintervention was 69% in the stented group versus 97% in the grafted group (*P* log-rank = .003; Figure 3, *C*).

### Survival

Eleven patients (10%) died during follow-up. Mode of death was cardiac in 2 patients, noncardiac in 3 patients, and unknown in 6 patients. Overall survival at 5, 10, and 14 years was 98%, 95%, and 80%, respectively (Figure 3, *D*). Survival was comparable to an age-, sex-, and race-matched reference population through 12 years and then decreased 12 to 14 years after primary coarctation repair.

## Discussion

### Principal Findings

**Figure 4:**
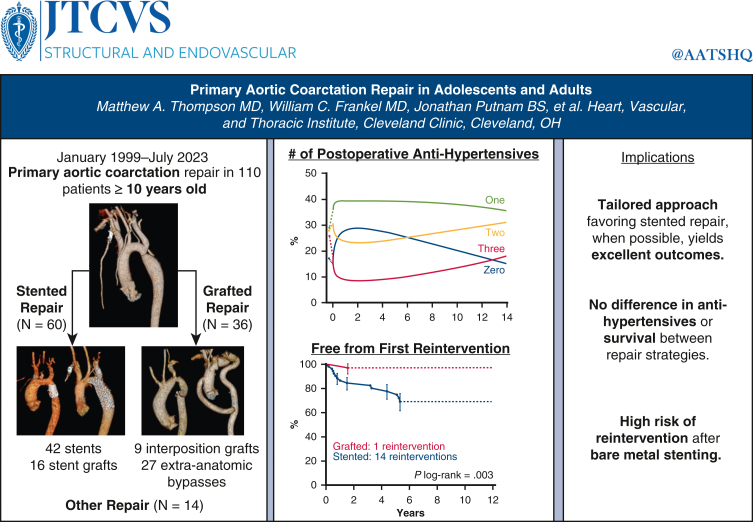
Graphical abstract.

1. In adolescent and adult patients, primary aortic coarctation repair can be achieved with excellent short-term outcomes using a variety of repair strategies, although long-term survival in this young cohort is lower than expected.
2. Coarctation repair may initially decrease antihypertensive requirements, but patients who are initially normotensive after surgery may still require antihypertensives years later.
3. Reintervention for in-stent restenosis is common after bare metal stenting, although they often can be achieved endovascularly with balloon angioplasty or repeat stenting (Figure 4).

### Selection of Repair Strategy

Adolescents and adults presenting with primary aortic coarctation are heterogeneous, and repair strategy must be tailored to their aortic anatomy while addressing concomitant cardiovascular lesions. Our current approach is to perform a stented repair with an endovascular balloon-expandable stent or stent graft in patients with amenable anatomy and without concomitant lesions. A single balloon-expandable stent is often adequate to expand the coarcted segment; however, addition of a second bare metal stent within a covered stent can facilitate technical success if the transcoarctation gradient remains high.

For stent graft repair, we typically use aggressive postdilation with a compliant balloon (Coda, Cook Medical). If necessary, dilation can be augmented with a stiffer balloon. None of our patients experienced an aortic rupture with this technique because the aortic fracture is already treated by the presence of the stent graft. However, it is vital to ensure that the stent graft lies parallel within an adequate length (>1.5 cm) of the proximal landing zone. The minimum aortic arch diameter should be 16 mm in women and 18 mm in men to minimize residual gradient through a hypoplastic arch. If the arch is more hypoplastic than that, we may perform a frozen elephant trunk with patch angioplasty to augment the proximal landing zone. In many cases, a zone 2 thoracic endovascular aortic repair, rather than a zone 3 repair, must be performed because of the proximity of the coarctation to the left subclavian artery orifice. Historically, we performed a staged repair with left carotid–subclavian bypass before zone 2 repair to maintain flow to the left upper extremity. Now, we increasingly favor a branched endoprosthesis (Thoracic Branched Endoprosthesis, WL Gore & Associates) to perform zone 2 repair and left subclavian artery revascularization in a single endovascular operation.^4^ This is an effective strategy for most patients, excluding those with large left subclavian artery aneurysms.

In contemporary practice, indications for interposition grafting in adolescents and adults are increasingly rare. The most recent patient in this series underwent interposition grafting in 2017, but the majority were before 2010. Because it is performed through a left thoracotomy, the exposure is often inadequate to address concomitant cardiovascular lesions. Injury to intercostal vessels, dilated from years of collateralized flow, is a potentially serious complication circumvented through median sternotomy or endovascular techniques.

When additional cardiovascular procedures are required, we may perform a frozen elephant trunk to address multi-segment thoracic aortic disease.^5^ Otherwise, we perform extra-anatomic bypass, which effectively addresses the coarctation, permits concomitant procedures through median sternotomy, and avoids circulatory arrest and arch dissection.^6^ Isolated extra-anatomic bypass may be used in patients with hypoplastic aortic arch or pan-hypoplastic aorta for whom stenting is not feasible or interposition grafting from the left chest may not address all aortic pathology.^7^^,^^8^

We have not used patch angioplasty or end-to-end anastomosis to repair adolescent or adult coarctation since 2012. Regarding the former, the coarcted aortic segment is histologically abnormal at baseline, and up to 58% of patients who undergo patch angioplasty develop repair-associated aneurysms of their residual diseased aorta within 25 years.^9^^,^^10^ Regarding the latter, in contrast to neonates and children, extended end-to-end anastomosis using the native aorta is rarely feasible in older patients, especially adults, due to lack of tissue mobility.

### Hypertension Management

Regardless of repair strategy, antihypertensive requirements decreased for 2 years after surgery, then gradually increased throughout follow-up. In general, patients returned to their baseline antihypertensive requirements by 14 years after repair. Multiple studies have demonstrated that short-term improvements in antihypertensive requirements do not persist with longer follow-up.^11^, ^12^, ^13^, ^14^, ^15^ In most patients, hypertension occurs independently of recoarctation.^11^^,^^16^ Identified risk factors for late hypertension in the literature are age more than 10 years at the time of surgery, hypertension at the first postoperative visit, and paradoxical hypertension after coarctation repair.^17^^,^^18^ Repair strategy has not been associated with late hypertension or exercise hypertension in multivariable analyses.^14^^,^^17^^,^^19^

### Reinterventions and Survival

Most reinterventions were for in-stent restenosis within 5 years of bare metal stenting. Repair durability and timing of reintervention in our cohort were consistent with prior literature. In the Coarctation of Aorta Stent Trial (COAST), 5-year freedom from reintervention in patients treated with balloon-expandable bare metal stents was approximately 75%.^20^ Combined data from COAST and COAST II, in which patients were treated with covered stents, reported 1- and 5-year freedom from reintervention of 95% and 79%, respectively.^21^ Known risk factors for reintervention include younger age at repair, stent diameter less than 12 mm, coarctation diameter less than 6 mm, and aortic wall injury.^20^^,^^21^ Reinterventions are nearly always endovascular but may be mitigated altogether by using covered stents or stent grafts in the index operation to allow for complete expansion of the culprit lesion. Durability of extra-anatomic bypass and interposition grafting were excellent, with only 1 graft-related complication—graft infection—during follow-up. Others have reported similar durability and excellent survival after interposition grafting^22^^,^^23^ and extra-anatomic bypass.^6^^,^^24^ Lifelong imaging surveillance remains critical to monitor for in-stent restenosis, recoarctation, or aneurysm formation. In our previous series from 2013, 61% of patients had undergone prior coarctation repair, and most presented for reintervention 20 or more years later.^1^

Long-term survival remains a challenge in patients with aortic coarctation. By 14 years after repair, survival was lower than an age-, sex-, and race-matched reference population. Older age at operation is one of the only consistent risk factors for late mortality identified in the literature.^13^^,^^14^^,^^18^^,^^25^, ^26^, ^27^ In patients who undergo index repair at age 20 years or less versus more than 20 years, 30-year survival is 92% versus 68%.^26^ Compared with age- and sex-matched controls, mortality is 3.3 times higher in patients aged 16 years or more who have undergone prior coarctation repair.^27^ This may be due to irreversible left ventricular hypertrophy that precipitates congestive heart failure or irreversible remodeling of vascular beds due to long-standing hypertension in patients who undergo late coarctation repair. Whether repair strategy influences long-term survival remains unclear.^28^

### Limitations

We grouped patients into stented and grafted strategies to minimize intragroup heterogeneity in repair technique and maximize the number of patients included in the comparison. Within the stented group, however, we describe variability in number and type of devices used because we lacked sufficient patients to stratify by device type. Our longitudinal analysis of antihypertensives does not consider dosage, compliance, or adequacy of blood pressure control. We opted not to use office blood pressure measurements available in the medical record because they are dependent on environment, cuff sizing, and patient positioning—factors we could not control retrospectively.

## Conclusions

Primary aortic coarctation repair in adolescents and adults can be performed with low mortality and, with advanced methods, low morbidity. Patients with isolated coarctation and adequate zone 2 diameter who do not require concomitant cardiovascular procedures are candidates for stented repair, although our data support abandoning bare metal stents given their high risk of in-stent restenosis. Those who do not meet these criteria can generally be treated with extra-anatomic bypass or frozen elephant trunk. Long-term outcomes are complicated by the persistent need for antihypertensives and long-term survival that is lower than expected in a young cohort. Early detection by a simple routine screening measurement in childhood of arm and leg blood pressure may facilitate early repair and improve long-term survival.

### Webcast

You can watch a Webcast of this AATS meeting presentation by going to: https://www.aats.org/resources/all-roads-lead-to-flow-open-hy-7310.

**Figure:**
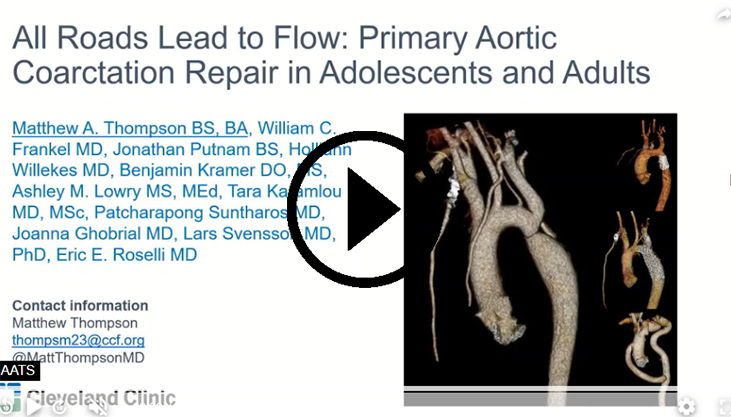

## Conflict of Interest Statement

E.E.R. is a consultant and speaker for Artivion, Cook Medical, Edwards Lifesciences, WL Gore & Associates, Medtronic, and Terumo Aortic. All other authors reported no conflicts of interest.

The *Journal* policy requires editors and reviewers to disclose conflicts of interest and to decline handling or reviewing manuscripts for which they may have a conflict of interest. The editors and reviewers of this article have no conflicts of interest.

## Figures and Tables

**Table E1:** Cardiovascular surgical history

Characteristic	n∗	Summary (N = 110)
No. of prior surgeries	110	
1		16 (15)
2		1 (0.9)
3		1 (0.9)
Hospital site	21†	
Cleveland clinic		4 (19)
Outside hospital		17 (81)
Procedure type‡		
Aortic valve		8
Aortic root		1
Ascending aorta		5
Descending thoracic aorta		1
Mitral valve		2
CABG		1
Other		11

*CABG*, Coronary artery bypass grafting.

∗ Number with data available.

† Eighteen patients underwent 21 cardiovascular operations preceding index coarctation repair.

‡ Procedure type not mutually exclusive.

**Table E2:** Patient characteristics stratified by operative approach

Characteristic	Stented (N = 60)		Grafted (N = 36)		*P*
	n∗	Summary	n∗	Summary	
Comorbidities					
Age (y)	60	36 ± 16	36	44 ± 14	.007
Male	60	36 (60)	36	21 (58)	.9
Hypertension	60	54 (90)	36	32 (89)	.9
No. of antihypertensives	60		35		.6
0		12 (20)		4 (11)	
1		16 (27)		12 (34)	
2		18 (30)		9 (26)	
≥3		14 (23)		10 (29)	
Smoking	60	17 (28)	36	12 (33)	.6
Dyslipidemia	60	7 (12)	36	15 (42)	<.001
Atrial fibrillation	60	4 (6.7)	36	2 (5.6)	>.9
Congestive heart failure	60	4 (6.7)	36	5 (14)	.3
Coronary artery disease	60	4 (6.7)	36	5 (14)	.3
Myocardial infarction	60	1 (1.7)	36	5 (14)	.03
Stroke	60	3 (5.0)	36	2 (5.6)	>.9
Diabetes	60	3 (5.0)	26	2 (4.6)	>.9
Cardiovascular morphology					
Concomitant congenital cardiac anomalies	60	39 (65)	36	25 (69)	.6
Bicuspid aortic valve		27 (45)		18 (50)	.6
Hypoplastic aortic arch		9 (15)		4 (11)	.8
Patent ductus arteriosus		3 (5.0)		2 (5.6)	>.9
Ventricular septal defect		2 (3.3)		2 (5.6)	.6
Hypoplastic abdominal aorta		1 (1.7)		2 (5.6)	.6
Pan-hypoplastic aorta		1 (1.7)		2 (5.6)	.6
Zone 1 diameter (mm)	34	24 ± 3.8	27	25 ± 6.4	.8
Zone 2 diameter (mm)	39	21 ± 4.0	26	23 ± 4.6	.11
Left subclavian artery diameter (mm)	42	14 ± 4.5	28	15 ± 3.9	.4
Concomitant thoracic aortic aneurysm	59	5 (8.5)	36	11 (31)	.005

∗ Number with data available.
